# Disentangling Molecular Links Underlying the Heart‐to‐Brain Connection in Older Adults

**DOI:** 10.1002/alz.085709

**Published:** 2025-01-09

**Authors:** Anna M. VandeBunte, Rowan Saloner, Emily W. Paolillo, Alexander J. Ehrenberg, Claire J. Cadwallader, Coty Chen, Tony Wyss‐Coray, Julio C. Rojas, Brandon Chan, Argentina Lario Lago, David A. Bennett, Joel H. Kramer, Kaitlin B. Casaletto

**Affiliations:** ^1^ Memory and Aging Center, UCSF Weill Institute for Neurosciences, University of California, San Francisco, San Francisco, CA USA; ^2^ Memory and Aging Center, Weill Institute for Neurosciences, University of California San Francisco, San Francisco, CA USA; ^3^ University of California, Berkeley, Berkeley, CA USA; ^4^ Stanford University, Stanford, CA USA; ^5^ Memory and Aging Center, Weill Institute for Neurosciences, University of California, San Francisco, San Francisco, CA USA; ^6^ Memory and Aging Center, UCSF Weill Institute for Neurosciences, University of California San Francisco, San Francisco, CA USA; ^7^ Rush Alzheimer's Disease Center, Rush University Medical Center, Chicago, IL USA

## Abstract

**Background:**

Cardiovascular disease (CVD) is among the strongest modifiable risk factors for dementia. However, vascular health is multifaceted, and its neurobiological underpinnings are unclear. A recent study (Williams et al. 2022) leveraged blood proteomics and machine learning to identify a novel group of 27 proteins that robustly predicted 4‐year likelihood of myocardial infarction, stroke, heart failure, or death. We examined whether these CVD‐related blood proteins also relate to brain health outcomes in older adults across three study cohorts.

**Method:**

Discovery cohort included 84 older adults from the UCSF Memory and Aging Center (57% female; 79% clinically normal; 21% mild cognitive impairment [MCI] due to Alzheimer’s disease [AD]) with plasma assayed for the 27 CVD‐related proteins (SomaScan) and plasma neurofilament light (NfL, Simoa). Participants completed brain MRI and neuropsychological evaluations. Separate regression models related each CVD protein to each brain health outcome (NfL, total gray matter volume, global cognition), adjusting for age, sex, BMI, and TIV and education, as appropriate. CVD proteins that reached significance in the discovery cohort were validated in an independent in‐vivo cohort with plasma SomaScan and all three brain outcomes (Stanford ADRC; n=40, 25% AD) and an autopsy cohort with brain tissue protein quantification (Selected Reaction Monitoring) and cognitive data (Rush ADC; n=1103, 40% AD) via parallel regression models.

**Result:**

In the discovery cohort, three CVD‐related plasma protein levels individually associated with global cognition (JAM2, PTPRJ, NCAM1, ps<0.03) and four proteins associated with plasma NfL (PTPRJ, GOLM1, SPON1, LRP11, ps<0.02). Only ADAMTS13 associated with total gray matter volume (p<0.01). In validation analyses, higher abundance of plasma SPON1 related to higher plasma NfL (β=0.38, p=0.02 [Stanford]), and higher abundance of SPON1 in brain tissue related to poorer cognition (β=‐0.13, p<0.0001 [Rush]). Brain tissue SPON1 also significantly differed across clinical diagnostic groups (normal≤ AD/MCI<AD dementia; f=4.71, p=0.0009).

**Conclusion:**

We demonstrate overlap in proteins associated with CVD risk and brain aging. Cross‐validation analyses in plasma and postmortem brain tissue highlight increased SPON1 as a robust predictor of worse neurobehavioral outcomes. SPON1 is an extracellular matrix protein involved in both smooth muscle and nervous tissue growth, and may represent a common link between heart health and brain health.

